# Supplementary material for: Production of H2-Free Carbon Monoxide from Formic Acid Dehydration: The Catalytic Role of Acid Sites in Sulfated Zirconia
Source: Nanomaterials (Basel). 2022 Sep 1;12(17):3036. doi: 10.3390/nano12173036 (PMC9458092; doi:10.3390/nano12173036)
Supplement: Supplementary file 1 [file nanomaterials-12-03036-s001.zip › nanomaterials-1893956-SI.pdf]

Supplementary Materials

Production of H_2-Free Carbon Monoxide from Formic Acid Dehydration: The Catalytic Role of Acid Sites in Sulfated Zirconia

Hyun Ju Lee [1,†], Dong-Chang Kang [2,†], Eun-Jeong Kim [3], Young-Woong Suh [4], Dong-Pyo Kim [2], Haksoo Han [1,*], Hyung-Ki Min [5,*]

[1] Department of Chemical and Biomolecular Engineering, Yonsei University, Seoul 03722, Korea.

[2] Department of Chemical Engineering, Pohang University of Science and Technology (POSTECH), Pohang 37673, Korea.

[3] School of Energy and Chemical Engineering, Ulsan National Institute of Science and Technology (UNIST), Ulsan 44919, Korea.

[4] Department of Chemical Engineering, Hanyang University, Seoul 04673, Korea.

[5] LOTTE Chemical Research Institute, Daejeon 34110, Korea.

* Correspondence: hshan@yonsei.ac.kr (H.H.); pulcherrima7@gmail.com (H.-K.M.)

†These authors contributed equally to this work.

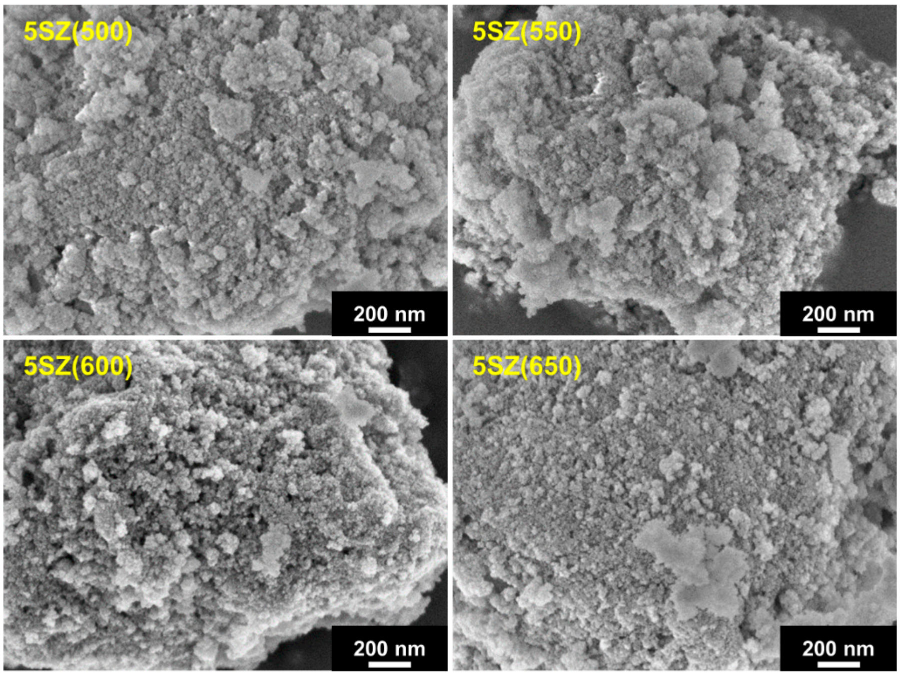

Figure S1. Scanning electron microscope (SEM) images of 5SZ(*y*) catalysts.

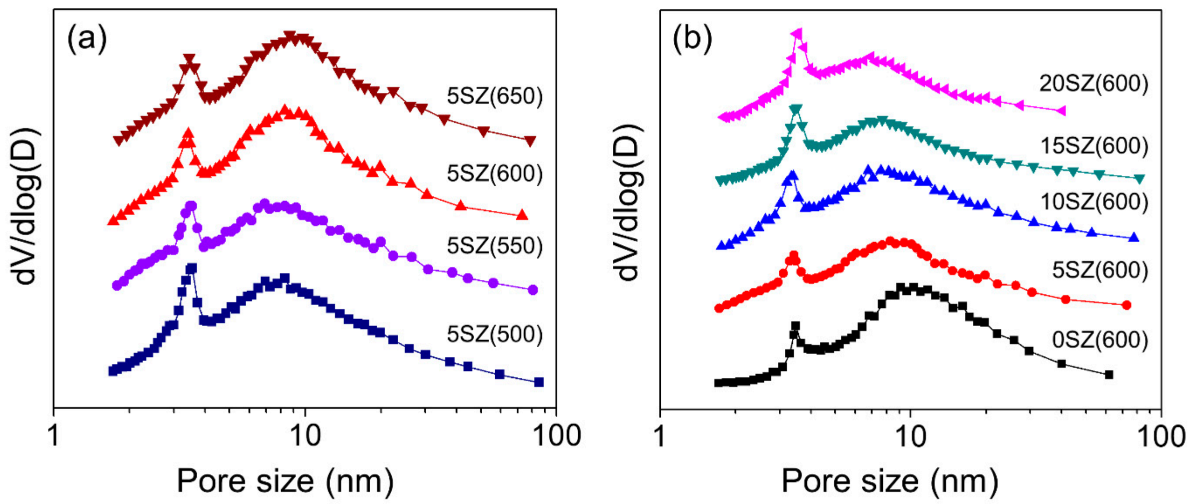

Figure S2. Pore size distribution of (a) 5SZ(*y*) and (b) *x*SZ(600) catalysts calculated from the desorption branch.

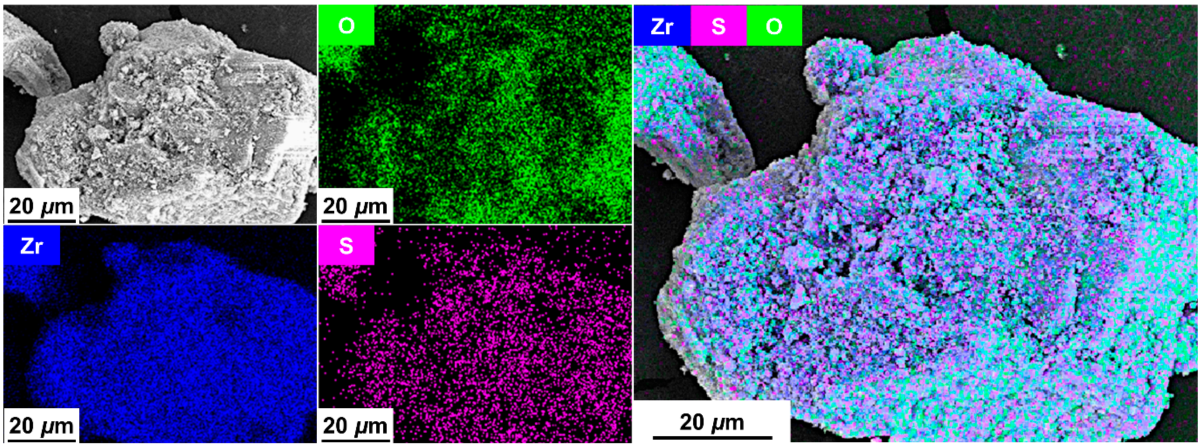

Figure S3. Scanning electron microscope–energy dispersive X-ray spectroscopy (SEM-EDS) images of 5SZ(600) catalyst.

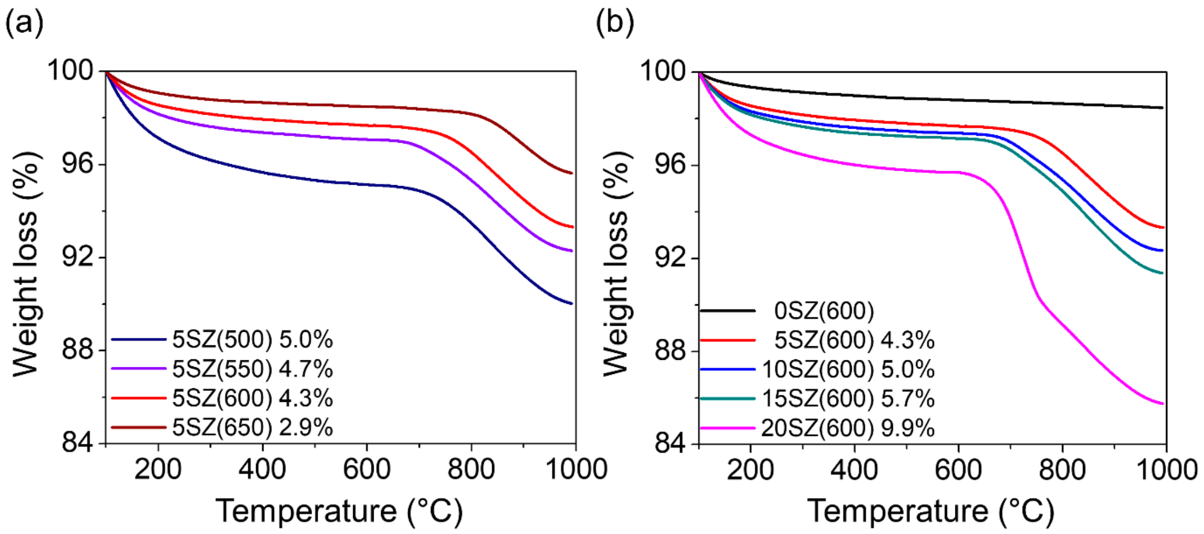

Figure S4. Thermogravimetric analysis profiles of (a) 5SZ(*y*) and (b) *x*SZ(600) catalysts.

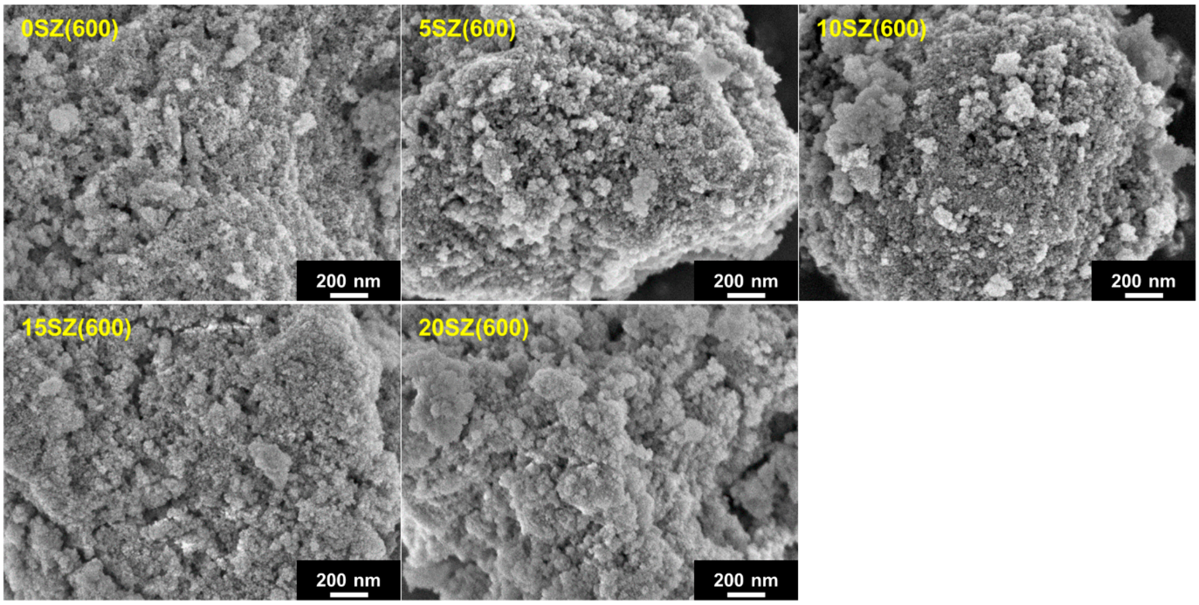

Figure S5. SEM images of *x*SZ(600) catalysts.

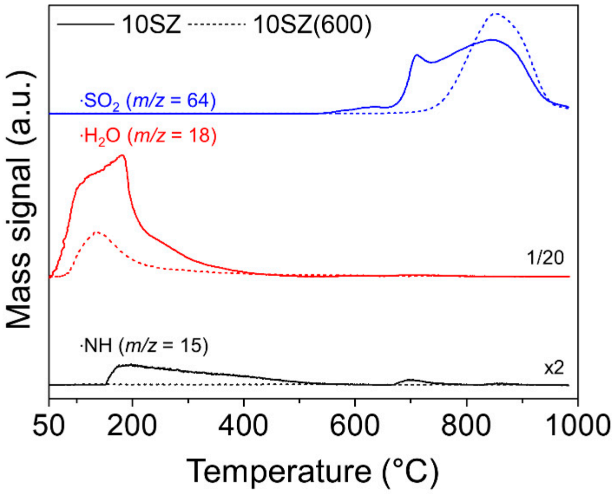

Figure S6. Evolution of ·NH (*m/z* = 15), ·H_2O (*m/z* = 18), and ·SO_2 (*m/z* = 64) in mass spectra as a function of temperature during temperature-programmed decomposition of as-prepared 10SZ (solid line) and calcined 10SZ(600) (dashed line) catalysts.

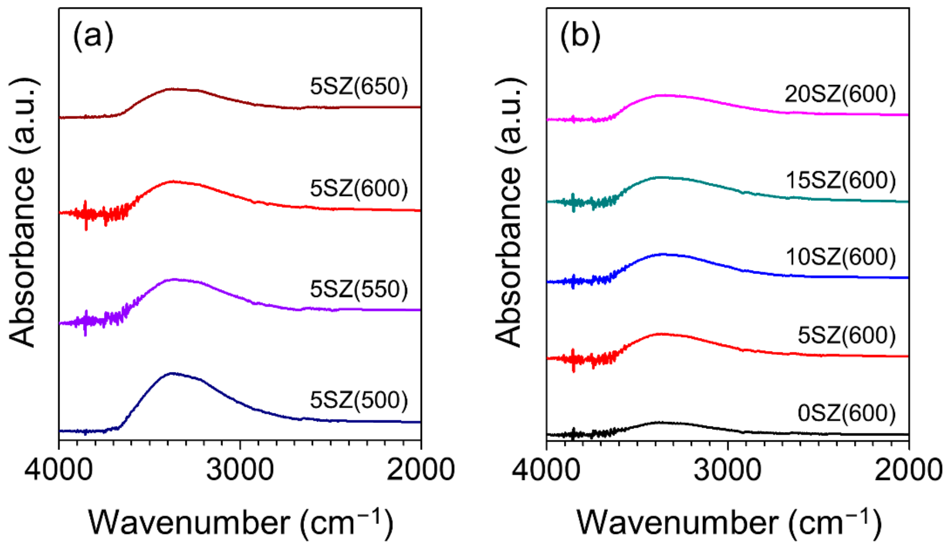

Figure S7. FT-IR spectra of (a) 5SZ(*y*) and (b) *x*SZ(600) in the range of 2000–4000 cm^{-1}.

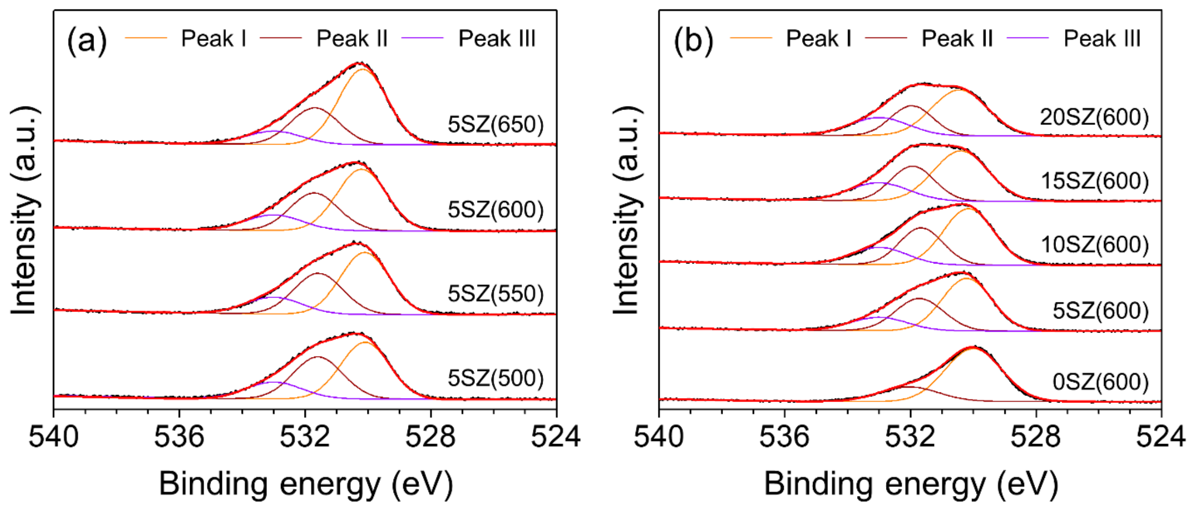

Figure S8. Deconvoluted O 1s XPS spectra for (a) 5SZ(*y*) and (b) *x*SZ(600) catalysts.

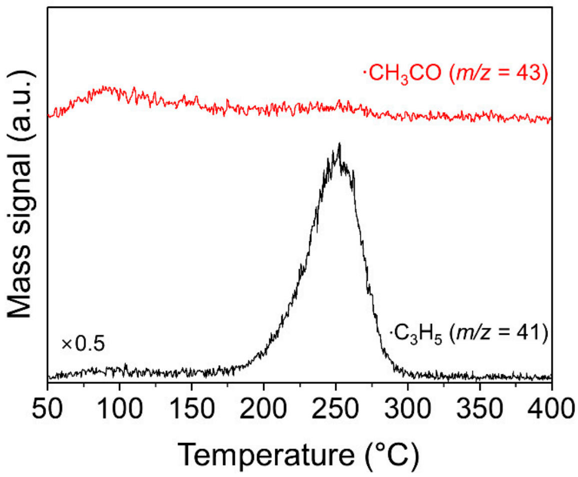

Figure S9. Evolution of ·CH_3CO (m/z = 43) and ·C_3H_5 (m/z = 41) in mass spectra as a function of temperature during IPA-TPD of 0SZ(600).

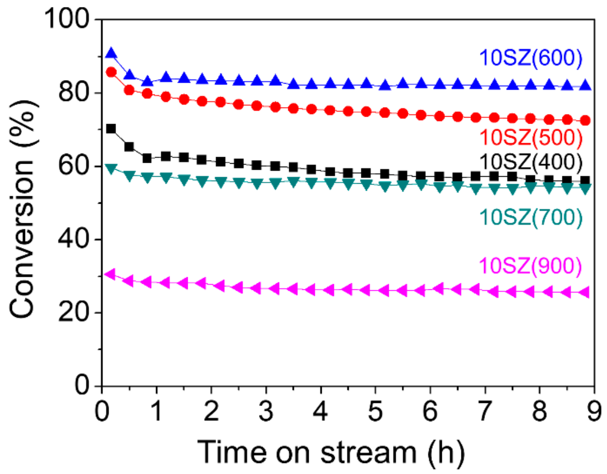

Figure S10. Formic acid conversion as a function of time on stream over 10SZ(*y*) catalysts at 260 °C and 6.0 h^{-1} WHSV.
